# Low-dose fixed-target serial synchrotron crystallography

**DOI:** 10.1107/S2059798317002996

**Published:** 2017-03-31

**Authors:** Robin L. Owen, Danny Axford, Darren A. Sherrell, Anling Kuo, Oliver P. Ernst, Eike C. Schulz, R. J. Dwayne Miller, Henrike M. Mueller-Werkmeister

**Affiliations:** a Diamond Light Source, Harwell Science and Innovation Campus, Didcot OX11 0DE, England; b Department of Biochemistry, University of Toronto, King’s College Circle, Toronto, Ontario M5S 1A8, Canada; c Department of Molecular Genetics, University of Toronto, King’s College Circle, Toronto, Ontario M5S 1A8, Canada; d Max Planck Institute for the Structure and Dynamics of Matter, Luruper Chaussee 149, 22761 Hamburg, Germany; e Departments of Chemistry and Physics, University of Toronto, King’s College Circle, Toronto, Ontario M5S 1A8, Canada

**Keywords:** macromolecular crystallography, serial synchrotron crystallography, fixed target, room-temperature crystallography, crystallography on a chip, low dose

## Abstract

The development of serial crystallography has been driven by the sample requirements imposed by X-ray free-electron lasers. Serial techniques are now being exploited at synchrotrons. Using a fixed-target approach to high-throughput serial sampling, it is demonstrated that high-quality data can be collected from myoglobin crystals, allowing room-temperature, low-dose structure determination. The combination of fixed-target arrays and a fast, accurate translation system allows high-throughput serial data collection at high hit rates and with low sample consumption.

## Abstract 2

A time- and sample-efficient approach for the serial collection of room-temperature diffraction data using a fixed target at a synchrotron is demonstrated.

## 1. Introduction   

X-ray free-electron lasers have driven the emergence of serial crystallography in recent years. The short duration and high intensity of X-ray pulses from a free-electron laser mean that protein crystals are destroyed by a single pulse. The short duration (5–50 fs) and high intensity (>10^12^ photons) of the pulses have the benefit that useful data can be collected before the crystal is destroyed (Chapman *et al.*, 2011 ▸), although the destructive nature of the interaction introduces the challenge of presenting a new crystal to each X-ray pulse. In order to address this challenge, a variety of sample-delivery methods have been developed, including, but not limited to, liquid jets, fixed-target arrays and goniometer-based approaches (all recently summarized by Chavas *et al.*, 2015 ▸).

The availability of tens of thousands of near-identical microcrystals has presented new opportunities and driven a desire to perform experiments using serial approaches (*i.e.* the rapid collection of data from many hundreds or thousands of crystals) at synchrotrons. Serial synchrotron crystallography (SSX) is attractive as the deleterious effects of radiation damage can be partially ignored despite the long duration of exposure to X-rays in comparison to an XFEL pulse. This is because the total dose required for structure determination is divided over a large number of crystals, with each crystal being subjected to an extremely limited dose. Cryocooling greatly slows the rate of radiation damage (Garman, 2010 ▸), allowing complete data sets to be collected from a single crystal or a small number of crystals, but even at cryotemperatures site-specific damage occurs extremely rapidly. This can lead to the presence of artefacts or ambiguity in the derived electron-density maps and is a particular challenge for metalloproteins, where the active site is often especially susceptible to X-ray-induced changes (Pearson & Owen, 2009 ▸).

While cryocooling does alleviate many of the problems associated with X-ray-induced radiation damage, data collection at room temperature can be preferable as cryocooling can hide conformational diversity (Fraser *et al.*, 2011 ▸) or introduce structural artefacts (Juers & Matthews, 2004 ▸). Furthermore, in the case of time-resolved crystallography, cryocooling can slow down or prevent turnover and hence preclude the observation of intermediates. The rapid decay of crystals held at room temperature provides an additional challenge, however, and usually requires data to be merged from many crystals; SSX provides a high-speed, high-throughput means of achieving this.

A number of approaches have been used to realise SSX, ranging from helical scans over crystals held within traditional cryoloops (Gati *et al.*, 2014 ▸) to a stream of crystals in a capillary (Stellato *et al.*, 2014 ▸). Other approaches include rastering over crystals held in random positions on a thin silicon wafer (Coquelle *et al.*, 2015 ▸), the use of an LCP extruder (Botha *et al.*, 2015 ▸; Nogly *et al.*, 2015 ▸) or more manual approaches in which crystals on a mount are individually aligned by hand (Murray *et al.*, 2015 ▸; Roedig *et al.*, 2016 ▸; Axford *et al.*, 2014 ▸). The manual centring of individual crystals on fixed targets provides a high hit rate (324 images from 324 crystals in the case of Murray and coworkers; five rotation data sets from five crystals in the case of Roedig and coworkers) and maximizes the data that can be collected from a limited number of crystals. A major drawback is that this results in low throughput, and manual centring of individual crystals is time-consuming. The low background scatter and high data quality obtained in both cases, however, highlight the potential of the approach.

We here demonstrate SSX using a fixed-target approach (Zarrine-Afsar *et al.*, 2012 ▸; Mueller *et al.*, 2015 ▸). Many thousands of crystals are held at known locations on a silicon chip, which is rapidly scanned through the X-ray beam in a few minutes. The chips and motion hardware and software are identical to those used successfully for SFX at LCLS and SACLA, demonstrating an approach suitable for use at both synchrotrons and XFELs. Diffraction data are collected in an efficient manner both in terms of the beamtime used and the number of crystals required to obtain a high-quality data set.

## 2. Materials and methods   

### 2.1. Sample preparation and chip loading   

Single-crystalline silicon chips provide an attractive means of achieving fixed-target crystallography with crystals held in precisely defined wells or features. We here use chips comprising 9 × 9 compartments, with each compartment made up of 12 × 12 bottomless apertures, or ‘features’, each of which is designed to catch and hold a crystal. The feature sizes are chosen to match the size of the crystals of the system under study. A small number of apertures are replaced with fiducial markers, meaning that a chip has a nominal capacity of 11 259 crystals [for further details of the fabrication and sample-loading process, please see Mueller *et al.* (2015 ▸) and Oghbaey *et al.* (2016 ▸)]. A chip is approximately 28 × 28 mm in size.

Orthorhombic sperm whale myoglobin (SWMb-CO) crystals were obtained as described in Oghbaey *et al.* (2016 ▸). Hexagonal sperm whale MetMb (SWMetMb) crystals were obtained in a similar way except that 10 m*M* potassium ferricyanide(III) was added to the gel-filtration peak fractions. After 10–15 min incubation, the protein was rapidly applied onto a Sephadex G25 column equilibrated with 10 m*M* Tris–HCl buffer pH 9.0. The eluate containing SWMetMb was concentrated to 55–60 mg ml^−1^ for crystallization. 180 µl crystallization solution containing the protein at 12–13 mg ml^−1^ in 10 m*M* Tris–HCl pH 9.0, 2.5–2.6 *M* ammonium sulfate was added to a Monoject Blood Collection Tube (10.25 × 64 mm, Covidien). 10 µl of SWMetMb crystal seeds was then added to the tube to induce crystallization. The crystals grew to approximately 50–80 µm in size before chip loading. In line with previous work (Mueller *et al.*, 2015 ▸), we found that the combination of (cuboid-like) crystals of <100 µm and the turbulent loading process resulted in a random distribution of crystal orientations across the chip, allowing complete data to be collected.

Chips were loaded with ∼50–80 µl of a crystalline suspension of SWMb-CO or SWMetMb crystals in a humidity-controlled environment (>50–60% relative humidity) to prevent salt-crystal formation on the chip from the high salt concentration in the mother liquor. Immediately after loading, the chips were sealed with 6 µm thick Mylar and mounted within a frame, allowing easy mounting onto the beamline *via* a kinematic mount. The loading process is straightforward and takes less than 2 min per chip, making the approach well suited for samples prepared *via* batch crystallization.

### 2.2. Data collection   

**Figure 1:**
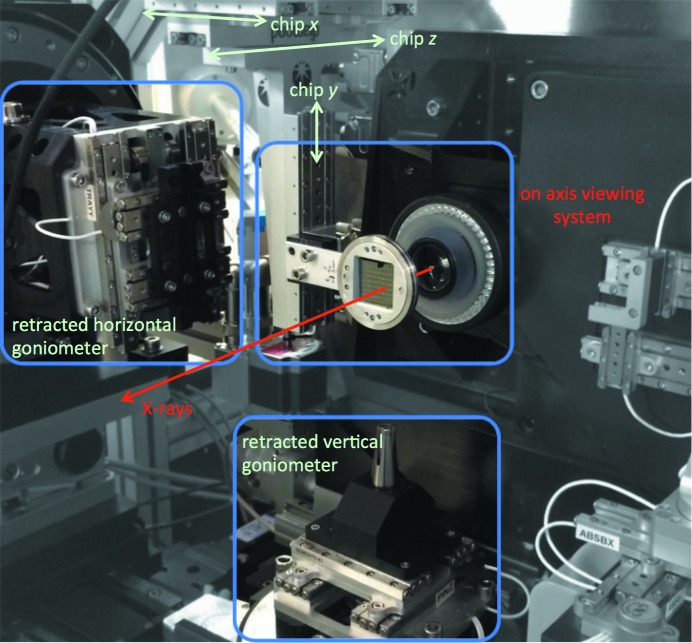
Instrumentation for fixed-target serial crystallography *in situ* on beamline I24 at Diamond Light Source. Retraction of both ‘standard’ goniometers leaves an empty sample environment, allowing the straightforward mounting of hardware for serial crystallography.

Diffraction data were collected on beamline I24 at Diamond Light Source. For serial data collection the standard pin and *in situ* goniometers were retracted to leave an empty sample environment. This allowed straightforward mounting of the portable stages described in Sherrell *et al.* (2015 ▸). The stages were inverted compared with the orientation previously used (Fig. 1 ▸), but otherwise motion and control was performed as described previously. The compact and modular nature of the setup was exploited and the existing beamline visualization, lighting, shuttering and attenuation were used.

A coordinate system is generated for every chip after mounting using three corner fiducials for alignment. The DeltaTau Geobrick LV-IMS-II motion controller is then able to rapidly move any aperture on a chip into the path of the beam by moving all three motors simultaneously and staying in the reference frame of the silicon-aperture array. This low-level motion-control environment moves the chip to a new location for each new frame of the detector in an accurate and time-efficient manner. This approach also allows diffraction images, and hence crystal parameters, to be linked to physical positions on the silicon chip, permitting two-dimensional occupancy maps to be drawn (see §[Sec sec3]3).

Communication and synchronization with the beamline equipment was achieved with a single TTL pulse sent from the chip-stage motion controller to the beamline ZEBRA logic and triggering controller (Quantum Detectors; http://quantumdetectors.com/zebra/) whenever a requested position was reached. The ZEBRA controller uses a series of logic commands to pass signals to beamline shutters and the detector as required. For user operation, a custom EPICS-based GUI was used to call all Python scripts required for data collection, move beamline equipment and allow straight­forward data collection. The GUI was also used to align chips and define the coordinate-system transform outlined above, allowing rapid and accurate motion in the coordinate frame of the chip.

Diffraction data were collected using two regimes, both at room temperature (294 K). In the first a beam size of 20 × 20 µm (*i.e.* matching the size of the aperture on a chip) was used with an incident flux of 1.4 × 10^12^ photons s^−1^ and an exposure time of 40 ms. In the second regime a beam size of 9 × 8 µm, an incident flux of 3.2 × 10^12^ photons s^−1^ and an exposure time of 25 ms were used. This resulted in absorbed doses of 51 and 426 kGy per crystal in the first and second regimes, respectively [absorbed doses were calculated using *RADDOSE* (Paithankar *et al.*, 2009 ▸) and data-collection parameters are summarized in Table 1 ▸]. Crystals were not rotated during an exposure, and data were recorded at an energy of 12.8 keV using a PILATUS3 6M detector. Crystal-to-detector distances of 340 mm (1.8 Å at the edge of the detector) and 305 mm (1.67 Å) were used in regimes 1 and 2, respectively.

### 2.3. Data analysis   

Diffraction frames were indexed and integrated using *DIALS* (Waterman *et al.*, 2016 ▸); the data were then scaled and merged in *PRIME* (Uervirojnangkoorn *et al.*, 2015 ▸). Three cycles of scaling and merging were used, with each cycle identifying the best subset of frames for use in the subsequent cycle (scaling statistics can be found in Table 1 ▸). Initial runs of merging and scaling in *PRIME* with default parameters gave output with pathologies suggestive of crystal twinning. This is most clearly observed in the variation of the second intensity moment for acentric data as a function of resolution (the results of intensity-moment analysis are tabulated as a function of resolution in the output of both *PRIME* and the latest version of *Phaser*; Randy Read, personal communication). For untwinned data, theoretical values of ∼2 are expected for the intensity moment, while initially we observed values that were closer to 1.5. The data were reprocessed using a less stringent sigma cutoff (sigma min -3), allowing negative intensities to be accepted into the final merged data set. This resulted in data sets with the expected second-order intensity moments. No significant change in scaling parameters such as *R*_merge_ or CC_1/2_, or *R* and *R*_free_ in the subsequent refinement, was observed between the data sets (<0.1%), although *I*/σ(*I*) decreased significantly in the outer resolution shells. The real-space *B* factor, however, showed considerable change, with mean values increasing from ∼9 Å^2^ to a more realistic ∼20 Å^2^. The resolution cutoff for each data set was defined as the point where CC_1/2_ fell to 0.5 in the outermost resolution shell. Structure solution was carried out by molecular replacement with PDB entry 1a6m (Vojtěchovský *et al.*, 1999 ▸) as a search model in *Phaser* (McCoy *et al.*, 2007 ▸). Refinement was carried out using *PHENIX* (Afonine *et al.*, 2012 ▸) and *Coot* (Emsley *et al.*, 2010 ▸).

## 3. Results   

A summary of the data-collection parameters and associated scaling statistics is shown in Table 1 ▸. The crystals on chip 1 were SWMb-CO in space group *P*2_1_2_1_2_1_, while the crystals on chip 2 were SWMetMb in space group *P*6. While a desire to minimize the absorbed dose (though defocusing and attenuating the X-ray beam in the case of chip 1 and reducing the exposure time in the case of chip 2) resulted in a relatively low signal-to-noise level, the indexing and scaling of data was not problematic and the data extended to 1.9 Å resolution or better in each case. The use of the second intensity moment as a criterion for including data resulted in the inclusion of more weak reflections, and we observed that while the overall data quality improved in the approach described in §[Sec sec2.3]2.3, this also contributed to a low signal-to-noise level. Anecdotally, the individual diffraction patterns did not appear to be significantly weaker than those collected during a comparable ‘standard’ oscillation experiment. Indeed, the aperture-like nature of each chip feature acts to reduce diffuse scatter on each image, meaning that background levels are low. An additional contribution to low signal-to-noise values may therefore be owing to the challenges associated with post-refinement and estimating the partiality of data from individual still images collected using a monochromatic beam.

**Figure 2:**
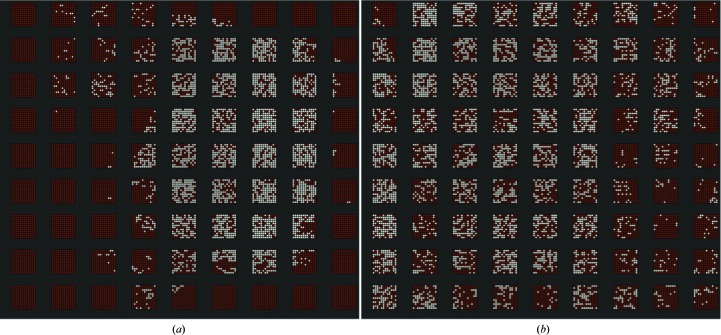
Chip maps showing the spatial distribution of crystals across chips for chip 1 (*a*) and chip 2 (*b*). Apertures resulting in an image successfully indexed with *DIALS* are shown in white; empty or unindexed apertures are shown in dark red.

For fixed targets of the pitch used (125 µm) the time taken to travel between positions is <10 ms. Even though this is an encoded move, we added a small dwell time (1 ms) at each position to ensure that the position is reached and the sample is stationary for data collection. These travel and dwell times mean that the total time taken to collect data from every position on a chip (11 664 locations, as this includes fiducial positions) was 10 min when 40 ms was used as the exposure time (chip 1) and 7 min when using an exposure time of 25 ms (chip 2). Two-dimensional maps illustrating the distribution of crystals across each chip are shown in Fig. 2 ▸. Chip 1 resulted in 2576 indexed images, representing a hit rate of 22%, while chip 2 resulted in 4264 indexed images (a hit rate of 37%). In both of the examples shown the hit rate is defined by how efficiently the chip is loaded. While recent work (Oghbaey *et al.*, 2016 ▸) has shown that for fixed targets hit rates can be significantly increased by characterization, or pre-mapping of occupied positions on chips, prior to X-ray data collection, this approach was not used in the experiments described here as the primary aim was to test and demonstrate other aspects of the experimental approach.

**Figure 3:**
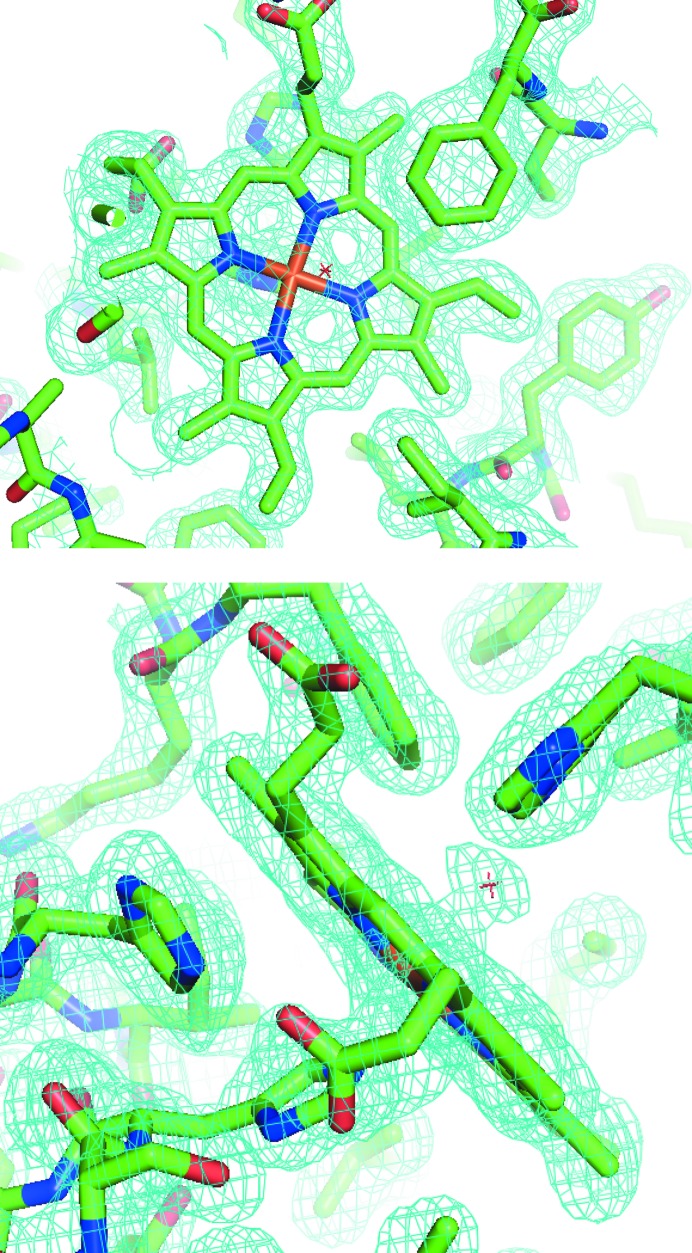
OMIT electron-density maps obtained from SW-MetMb crystals mounted on chip 2. The maps are contoured at 1σ and show density in the region of the porphyrin ring. Composite OMIT maps were calculated using *PHENIX* (Terwilliger *et al.*, 2008 ▸).

In each case sufficient data were obtained from a single chip for structure solution. For both crystal forms structure solution was straightforward, although the hexagonal data (chip 2) were affected by the possibility of ambiguous indexing (Brehm & Diederichs, 2014 ▸). This was resolved through the use of a reference rotation data set previously obtained on beamline I24. The derived electron-density maps and models from chips 1 and 2 are shown in Fig. 3 ▸.

## 4. Discussion   

The low-dose structure solution from myoglobin crystals mounted on silicon chips at room temperature demonstrates the realisation of SSX in a sample- and time-efficient manner. The method by which the chips are loaded means that data are collected from a large fraction of all crystals prepared. Furthermore, those not successfully loaded can be recovered for loading onto subsequent chips. The maps obtained from chip 1 show that even a modestly loaded chip provides sufficient data for structure solution. The exposure time per aperture defines the throughput of our approach, with a significant advantage being that this can be easily varied according to the available flux or crystals to hand. On beamline I24, for ‘well diffracting’ samples we find that 25 ms exposures are sufficient for successful data collection using still images; this means that data can be collected from all 11 664 positions on a chip in less than 7 min. The time taken to exchange and align chips is somewhat less than this, and will become greatly reduced when stages that are capable of holding multiple chips and automated chip exchange are developed. In our approach only one alignment step is needed: alignment of the chip. With a good-quality on-axis viewing system this is relatively straightforward and allows the collection of data from many thousands of crystals in a few minutes.

Radiation damage is an acute problem in room-temperature synchrotron crystallography. Metalloproteins and heavy-atom derivatives are particularly susceptible, as the presence of heavy atoms significantly increases the X-ray cross-section of the crystal (Owen & Sherrell, 2016 ▸) and the active site may be particularly susceptible to X-ray-induced changes. In all serial experiments the total dose required for structure solution is spread over the many crystals used. Here, in the low-dose SWMb-CO example, each crystal is subjected to a dose of 51 kGy, which is significantly less than the ‘safe dose’ of 80 kGy measured for these crystals using UV–Vis absorption spectroscopy (Oghbaey *et al.*, 2016 ▸). Serial approaches therefore provide a means of obtaining low-dose, low-damage room-temperature structures at synchrotrons. In the case of SWMetMb, site-specific damage is much more likely to be present (absorbed dose per crystal of 426 kGy). No obvious signs of X-ray-induced damage could be seen in the obtained electron density; however, this perhaps indicates that any changes are subtle and are best tracked using complementary methods such as UV–Vis, especially as they are likely to centre around the haem iron.

The ability to reliably relate each diffraction pattern to a specific crystal allows combination of the fixed-target approach with spectroscopic applications carried out prior or subsequent to the diffraction experiment. The use of spectroscopy before the X-ray experiment allows mapping approaches, while its use afterwards raises the possibility of quantifying the fraction excited in photo-activated experiments through comparison of spectral changes.

While relatively straightforward to implement, the collection of still (zero-oscillation) images is not optimal on a monochromatic synchrotron beamline. Despite using this approach, we show here that structure solution is straightforward with a relatively low number of integrated frames, well below the capacity of a single chip. Ideally, SSX approaches should be inspired by XFEL methodologies but not defined by the destructive power of XFELs, and tailored to the strengths and capabilities of a synchrotron beamline. In order to maximize the amount of data collected from each crystal, an oscillation should be introduced while the crystal is in the beam. This would result in data similar to those obtained by Gati *et al.* (2014 ▸) and would result in the collection of a single rotation image or a thin wedge of images from each crystal while preserving the high hit rates offered by fixed targets. The use of a fixed-target approach allows this to be achieved and will have the complementary benefits of reducing the number of crystals required for structure solution and simplifying data processing, as a small number of reflections will be fully recorded. On a practical level this is technically challenging, but initial work on beamline I24 suggests that synchronization of an air-bearing goniometer with the fast linear translation stages described is possible. In the presence of radiation damage, the additional frames could be exploited for post-refinement and partiality correction before being ignored during integration. Ongoing developments at synchrotrons such as the availability of non-monochromatic, or pink, beams may also be beneficial for serial approaches. The use of a pink beam will result in more reflections being recorded on each (still) image and reduced exposure times may be used, allowing higher temporal resolution to be achieved. These and other developments such as the upcoming generation of high-frame-rate detectors will result in improved data quality and increased throughput in the field of (fixed-target) serial synchrotron crystallography.

## Supplementary Material

PDB reference: SWMb-CO, 5m3r

PDB reference: SWMetMb, 5m3s

## Figures and Tables

**Table 1:** Data-collection parameters, scaling and refinement statistics Statistics for the outermost resolution shell are shown in parentheses. Ramachandran statistics were determined using *MolProbity* (Chen *et al.*, 2010 ▸).

	Chip 1 (SWMb-CO)	Chip 2 (SWMetMb)
Data collection		
Exposure time (ms)	40	25
Beam size (µm)	20 × 20	9 × 8
Wavelength (Å)	0.9686	0.9686
Incident flux (photons s^−1^)	1.4 × 10^12^	3.2 × 10^12^
Absorbed dose (kGy)	51	426
No. of integrated frames	2576	4264
No. of images used†	1776	3894
Scaling		
Space group	*P*2_1_2_1_2_1_	*P*6
Unit-cell parameters (Å)	*a* = 37.87, *b* = 46.86, *c* = 84.7	*a* = *b* = 91.44, *c* = 45.96
Resolution (Å)	42.35–1.80 (1.83–1.80)	39.76–1.70 (1.73–1.70)
*R*_merge_	0.795 (0.926)	0.761 (0.958)
*R*_split_	0.235 (0.663)	0.148 (0.514)
CC_1/2_	93.57 (47.5)	97.79 (58.87)
〈*I*/σ(*I*)〉	4.27 (0.36)	1.65 (0.23)
Multiplicity	25.3 (18.3)	75.2 (56.43)
Completeness (%)	100.0 (100.0)	99.8 (99.3)
Refinement		
No. of reflections	14556	24300
No. of non-H atoms		
Protein	1299	1246
Water	174	201
*R*/*R*_free_	0.196/0.248	0.180/0.225
R.m.s.d., bond lengths (Å)	0.008	0.006
R.m.s.d., bond angles (°)	0.917	0.858
Ramachandran plot		
Most favoured (%)	98.0	98.0
Allowed (%)	2.0	2.0
PDB code	5m3r	5m3s

† Images were primarily rejected from the scaling step owing to crystal-to-crystal variation in the unit-cell parameters.
